# Blurring of High-Resolution Data Shows that the Effect of Intrinsic Nucleosome Occupancy on Transcription Factor Binding is Mostly Regional, Not Local

**DOI:** 10.1371/journal.pcbi.1000649

**Published:** 2010-01-22

**Authors:** Wee Siong Goh, Yuriy Orlov, Jingmei Li, Neil D. Clarke

**Affiliations:** Computational and Systems Biology, Genome Institute of Singapore, Singapore; Weizmann Institute of Science, Israel

## Abstract

Genome wide maps of nucleosome occupancy in yeast have recently been produced through deep sequencing of nuclease-protected DNA. These maps have been obtained from both crosslinked and uncrosslinked chromatin in vivo, and from chromatin assembled from genomic DNA and nucleosomes in vitro. Here, we analyze these maps in combination with existing ChIP-chip data, and with new ChIP-qPCR experiments reported here. We show that the apparent nucleosome density in crosslinked chromatin, when compared to uncrosslinked chromatin, is preferentially increased at transcription factor (TF) binding sites, suggesting a strategy for mapping generic transcription factor binding sites that would not require immunoprecipitation of a particular factor. We also confirm previous conclusions that the intrinsic, sequence dependent binding of nucleosomes helps determine the localization of TF binding sites. However, we find that the association between low nucleosome occupancy and TF binding is typically greater if occupancy at a site is averaged over a 600bp window, rather than using the occupancy at the binding site itself. We have also incorporated intrinsic nucleosome binding occupancies as weights in a computational model for TF binding, and by this measure as well we find better prediction if the high resolution nucleosome occupancy data is averaged over 600bp. We suggest that the intrinsic DNA binding specificity of nucleosomes plays a role in TF binding site selection not so much through the specification of precise nucleosome positions that permit or occlude binding, but rather through the creation of low occupancy regions that can accommodate competition from TFs through rearrangement of nucleosomes.

## Author Summary

Genomic DNA is largely covered by proteins that compete with one another for binding to regulatory sequences. Most of these proteins are in the form of nucleosomes. How nucleosomes come to occupy particular sites and thereby compete with sequence specific transcription factors is a central problem in developing a systems-level understanding of gene regulation. Here, we performed a series of computational analyses using high-resolution nucleosome position data that has recently become available in yeast, thanks to advances in DNA sequencing technology. Analysis of these data, combined with data on the location and occupancy of transcription factors genome-wide, shows that the precise location of nucleosomes as determined by nucleosome sequence specificity is often less important to transcription factor binding than the broader, regional occupancy of nucleosomes that is encoded in genomic DNA. This result has implications for the evolution of DNA regulatory elements.

## Introduction

Genomic DNA is largely covered in proteins, mostly in the form of nucleosomes. Much of the remainder consists of chromatin-associated proteins, including enzymes that modify histones or DNA, or catalyze the rearrangement of nucleosomes, and sequence specific DNA binding proteins (transcription factors) that mediate the activation or repression of genes. A deep understanding of gene regulation requires an understanding of how each of these cooperate and compete for access to genomic DNA.

That nucleosomes and TFs do, in fact, compete on a genomic-wide scale was substantiated several years ago by chromatin immunoprecipitation microarray experiments (ChIP-chip), which determined the distribution of histones along the yeast genome. [1]–[3] These studies revealed an under-representation of nucleosomes in promoter regions, relative to transcribed regions. In contrast, TFs are under-represented in transcribed regions and enriched in promoter regions. Furthermore, nucleosome occupancy differs among promoters and these differences correlate with the probability of TF binding. [4]

Competition between nucleosomes and transcription factors is a simple consequence of each having an inherent probability of binding to the same site. Some transcription factors may be able to bind DNA on the outside surface of a nucleosome, but steric occlusion of sites on the inside and the sharp bending of DNA around the nucleosome preclude most transcription factors from binding to nucleosomal DNA. As a consequence of this competition, nucleosomes can mediate interactions among transcription factors (TFs) in an entirely passive way. For example, a binding motif that is close to a second, TF-occupied, site will tend to have a lower nucleosome occupancy than it would in the absence of the occupied site because nucleosome configurations that span both the motif and a nearby occupied site are disallowed. This lower nucleosome occupancy translates into a higher effective binding affinity of the site. In this scenario cooperative binding of factors is mediated not by direct interactions between the factors but by the passive effects of nucleosomes due to mutual competition. This effect has been demonstrated experimentally. [5]

Passive mediation of TF-TF interactions is one way in which nucleosomes affect the occupancy of TFs in the genome. A second is through the intrinsic sequence specificity of nucleosomes themselves. Nucleosomes lack highly specific amino acid side chain-to-base pair contacts that are characteristic of sequence-specific transcription factors, but they do have sequence preferences that are determined by the capacity of the DNA to be wrapped around the nucleosome. One manifestation of this preference is a subtle but significant tendency towards a 10bp periodicity of certain dinucleotide steps. [2], [6]–[9] This periodicity reflects the helical periodicity of DNA in nucleosomes and differences in the propensity for structural perturbations among different basepair-basepair steps. [10] More recently, attention has been drawn to longer A-rich regions, which are inhibitory for nucleosome binding [9],[11],[12]. The near-exclusion of these sequences from the central regions of nucleosomes was first noted around the same time that dinucleotide preferences were discovered [7] but it has become clear only recently that these sequences contribute substantially to the ability to predict nucleosome binding. [9],[11],[12]

Kaplan et al recently provided a simple and elegant demonstration that the intrinsic sequence specificity for nucleosome binding is a partial determinant of nucleosome positioning in vivo. [9] In their work, nucleosome positions were determined from deep sequencing of nuclease-digested chromatin prepared from yeast cells, and also from chromatin reconstituted in vitro from chicken histones and yeast genomic DNA. Sequence features associated with nucleosome occupancy were found to be similar in real chromatin and in reconstituted chromatin. Furthermore, genomic loci that are bound by transcription factors have, on average, lower nucleosome occupancies than unbound sites even when nucleosome occupancies are determined in vitro. This suggests that intrinsic nucleosome binding preferences have some effect on the selection of binding sites by transcription factors in vivo. Kaplan et al further reported that there are no significant differences in mapped nucleosome positions when chromatin was crosslinked compared to the more conventional procedure of mapping without crosslinking. [9] Mapping without crosslinking assumes that sites occupied by nucleosomes in vivo remain bound to those sites on the time scale of the assay. The reported similarity of the data using the two procedures suggests that that is largely the case.

Here, we revisit the data of Kaplan et al, analyzing it in the context of existing ChIP-chip data [13] as well as new, more quantitative ChIP-qPCR experiments reported here. We confirm a role for intrinsic nucleosome binding preferences in the binding of transcription factors, though our analyses suggest a rather low bound on how much information may be provided. More interestingly, we show that the role played by sequence specific nucleosome binding lies not so much in the precise positioning of nucleosomes as in the determination of the average nucleosome density over a promoter-sized region. We also show that there is a difference between crosslinked and uncrosslinked chromatin that is at least as informative with regard to the binding of transcription factors as either data set is alone.

## Results

### Low nucleosome occupancy at transcription factor binding motifs can be discerned even at sites with low transcription factor occupancy

**Figure 1:**
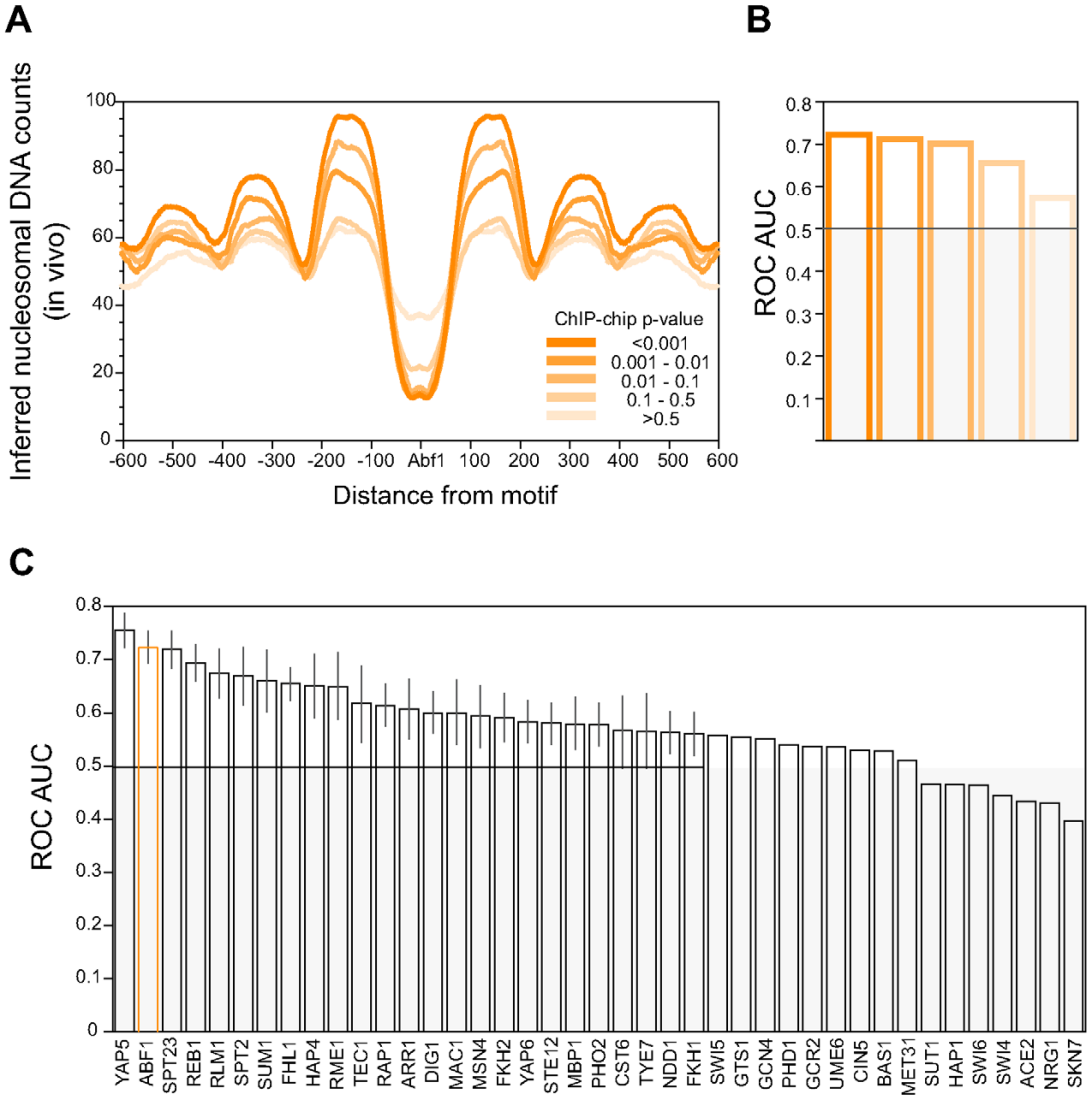
Nucleosomal DNA is depleted at transcription factor binding sites. (A) Nucleosomal tag counts in the vicinity of Abf1 motifs as a function of p-value for ChIP enrichment of the site. Abf1 sequence motifs, and the sites bound at p≤1e-3, were defined by MacIssac et al. [16] Abf1 motifs considered unbound by MacIssac et al (p>1e-3) were assigned the Harbison et al ChIP enrichment p-values of the genomic region spanning the site. [13] Only ChIP-enrichment values obtained in YPD media were used. (B) Area under ROC curves (ROC AUC) for the prediction of Abf1 binding based on low nucleosomal DNA tag counts. Abf1 bound sites were compared to randomly selected yeast promoter sites, using 15bp windows centered on the Abf1 and random sites. Values above 0.5 are considered significant. (C) ROC AUC values as in panel B but for a total of 41 transcription factors. Bound sites were defined as for the Abf1 sites using p-value for enrichment of 1e-3 or better. All transcription factors that had at least 50 bound sites are shown.

The analyses in this paper make extensive use of the nucleosome mapping data reported recently by Kaplan et al, so it was important to first establish that we can replicate and extend an analysis that they performed. To that end, we took Abf1 motifs in the yeast genome and calculated a set of averaged profiles of nucleosome sequence tags around those motifs (Figure 1A). The most prominent of these nucleosome profiles, showing a very deep minimum for occupancy at the Abf1 motifs, was calculated for Abf1 motifs that are bound by the protein with high confidence (ChIP-chip enrichment p-value≤1e-3). This particular profile likely corresponds to the single profile shown in Figure 4c of Kaplan et al, [9] though it has been calculated here over a wider genomic interval to show the enrichment of phased nucleosomes up to three units away from the Abf1 motifs.

We extended this analysis by testing other Abf1 sites for which the evidence for Abf1 binding is weaker. Remarkably, as the stringency for defining Abf1 binding is relaxed, from the most stringent (p≤1e-3) to the least (p>0.5), both the depletion of nucleosomes at the Abf1 motif and the enrichment of nucleosomes at adjacent flanking positions decrease, but not to the point where they disappear altogether (Figure 1A). This is reminiscent of the analyses of Tanay, who provided evidence for TF binding at ChIP enrichment p-values far worse than what would ordinarily be considered a meaningful threshold for binding. [14]

To assess quantitatively the correlation between low nucleosome occupancy and TF binding we asked how well nucleosome tag counts correctly distinguish TF-bound sites from random sites selected from yeast promoters. We use the area under the ROC curve (ROC AUC: receiver operator characteristic area under the curve) as a measure of this association. [15] As shown in Figure 1B, for even the lowest confidence Abf1 binding sites (p>0.5), the ROC AUC exceeds 0.5, the value that is expected by chance. This analysis was extended to the 41 yeast TFs for which there are at least 50 binding motifs bound according to the ChIP-chip data of Harbison et al (p≤1e-3) and the subsequent motif analysis of MacIsaac et al. [13],[16] Bootstrap analysis of ROC curve areas shows a significant association between TF occupancy and nucleosome depletion for most of the 41 TFs (Figure 1C).

### TF binding sites tend to be associated with excess nucleosome counts in crosslinked chromatin vs. uncrosslinked

Kaplan et al used two different methods in their nucleosome mapping experiments, one involving formaldehyde crosslinking (two replicates) and the other a more traditional non-crosslinking protocol (four replicates). [9] Crosslinking should be unnecessary if nucleosomes are sufficiently stable that nucleosomal locations are the same at the end of the assay as they are in vivo. Having performed the experiment both ways, Kaplan et al deemed the two data sets to be sufficiently similar that they averaged all six replicates and used this single set of averaged tag counts for their analyses. [9] However, they make available the two separate datasets.

For Figure 1 we used data from uncrosslinked chromatin only, but we performed the analysis with data from crosslinked chromatin as well. Our expectation was that nucleosome occupancies obtained from crosslinked chromatin would show, if anything, a stronger association with transcription factor binding than uncrosslinked chromatin because crosslinking would prevent the movement of nucleosomes into regions that are occupied by TFs in vivo but which might rearrange in the time course of the experiment. Surprisingly, we found that crosslinking generally weakened the association between TF binding and nucleosome depletion at TF sites, rather than strengthening it. (Figure S1)

**Figure 2:**
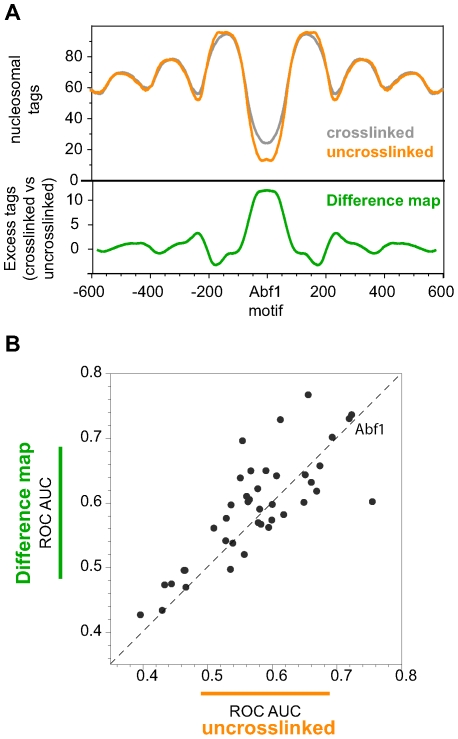
Crosslinking of chromatin preferentially protects sites that are otherwise nuclease sensitive and correlated with transcription factor binding. (A) (top): Tag counts of uncrosslinked chromatin (orange) and crosslinked chromatin (gray) in the region of bound Abf1 sites. Tag counts have been symmetrized around the Abf1 site. Tag counts for the crosslinked sample were normalized to the uncrosslinked sample between 100–600bp from the Abf1 site to highlight the concordance in the phased nucleosome locations and occupancies. (bottom): Tag count difference map (green) in the vicinity of bound Abf1 sites showing excess tags in crosslinked chromatin vs. uncrosslinked. (B) Predictive value of nuclease-resistant tag counts for binding of 41 TFs. ROC AUC values on the y-axis were calculated based on the difference map (excess tag counts found in the crosslinked sample compared to the uncrosslinked). ROC AUC values on the x-axis were calculated as in Figure 1. The dashed line is for y = x. Binding of most TFs is predicted by the difference map well as well as, or even better than, by the under-representation of tags in the normal chromatin preparation.

To investigate this result further, we examined the crosslinked tag count distribution around Abf1 sites, as was done for Figure 1A using the data from uncrosslinked chromatin. Inspection of the tag count distribution around bound Abf1 motifs reveals a remarkable concordance between the two data sets in the enrichment of nucleosome tag sequences flanking the binding site. These correspond to a series of (averaged) phased nucleosomes adjacent to Abf1 binding sites (Figure 2A). However, at the Abf1 site itself there is a reduction in the amount of nucleosome depletion inferred from the crosslinked chromatin. It is this slightly less dramatic effect that reduces the predictive value of nucleosome tags in predicting Abf1 binding sites when crosslinked data is used rather than uncrosslinked.

The bottom panel in Figure 2A shows the difference between the averaged tag counts in the crosslinked experiment and the averaged (and normalized) tag counts in the uncrosslinked experiment. A clear peak in excess tag counts can be seen at Abf1 sites, suggesting that excess tag counts found in the crosslinked experiment are associated with TF binding. To assess this more broadly, we normalized and subtracted, genome wide, the tag counts obtained with uncrosslinked chromatin from those obtained with crosslinked chromatin to produce a “nucleosome difference map”. We then asked whether high tag counts in the difference map could predict TF binding in a manner analogous to how low tag counts in the raw nucleosome maps predict TF binding. Remarkably, excess tags in the crosslinked chromatin are as strongly correlated with TF binding as nucleosome depletion is in the uncrosslinked sample (Figure 2B). This is reflected in the strong correlation between the two sets of ROC AUC values (R = 0.80) and in the slope of the line relating the values (∼1.02). If anything, the excess tags found in the crosslinked sample are more strongly correlated with TF binding than is nucleosome depletion in the uncrosslinked library alone. 26 out of 41 TFs have higher ROC AUC values based on excess crosslinked tags, and the absolute value of the differences for those 26 are about 50% higher on average than for the remaining 15. We suspect this effect is due to the crosslinking of nucleosomes that span the binding site. Crosslinking will trap transiently bound nucleosomes, and will likely do so more efficiently than for TFs because of the large number of amines (lysines and arginines) that lie in close proximity to DNA.

That crosslinking appears to be trapping nucleosomes over TF binding sites is illustrated by the appearance of a very strong nucleosome peak in the difference map that lies right on top of a set of Gal4 sites in the GAL1–GAL10 promoter (Figure S2). The nucleosome is present when crosslinked, and nearly absent when not; a set of six neighboring nucleosome positions are scarcely affected by crosslinking. The crosslinked nucleosome is much less prominent when cells are grown in galactose, presumably because Gal4 occupancy is higher under these conditions, resulting in less opportunity for a nucleosome to be crosslinked at that location.

Regardless of the mechanism, the association between TF bound sites and excess tag counts in crosslinked chromatin suggests that difference maps based on crosslinked and uncrosslinked chromatin might be used to identify non-histone DNA-binding sites without ChIP enrichment for particular proteins. How such sites would compare to DNase hypersensitive sites or nucleosome poor regions defined by FAIRE [17] remains to be seen.

### Effect of intrinsic chromatin on TF occupancies as determined by ChIP-qPCR of consensus binding sites

Kaplan et al. made the important observation that genomic loci that are bound by TFs in vivo tend to be also depleted for nucleosomes in reconstituted chromatin. [9] This shows that at least some of the low nucleosome occupancy observed at TF sites is intrinsic to the DNA binding specificity of nucleosomes and is not simply a consequence of competition by TF binding. As a prelude to our analysis of resolution-sensitivity, we validated the observations made by Kaplan et al using the same TF binding data but with a different analytical measure (ROC AUC vs. average tag counts). We also obtained additional, higher accuracy TF binding data using ChIP-qPCR at selected binding motifs in order to establish more quantitatively the correlation between binding and nucleosome occupancy.

**Figure 3:**
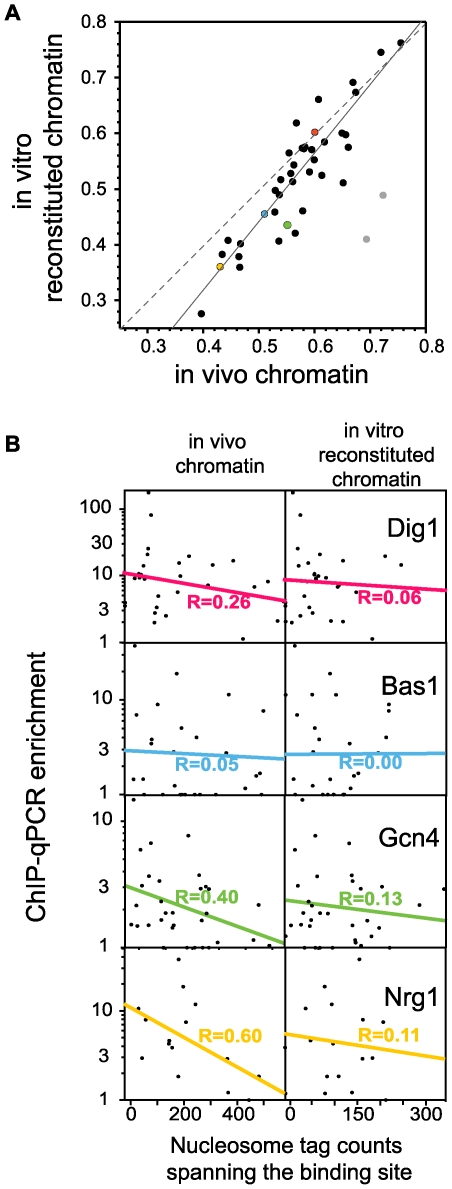
Some of the nucleosome position information correlated with transcription factor binding is intrinsic to genomic sequence. (A) ROC AUC values quantifying the predictive value of low nucleosome occupancy based on chromatin in vivo (x-axis) or chromatin reconstituted in vitro from genomic DNA and histones (y-axis). The in vivo data are as shown in Figure 1; values based on the in vitro reconstituted chromatin were calculated in the same manner. The positions of four TFs that are used in panel B are indicated by colored circles. The solid line is the best linear fit through the data (R = 0.90), excluding outliers Abf1 and Reb1 (gray circles). That Abf1 and Reb1 are truly outliers was established by assessing the deviation from a fit to all the data: these two TFs deviate from that line by a distance that exceeds the average distance by more than 2.5 standard deviations. The dashed line, y = x, corresponds to the expected fit if in vitro and in vivo nucleosome data were entirely equivalent. (B) Correlation between the ChIP enrichment value at perfect consensus binding sites and tag counts from nuclease-protected mono-nucleosome-sized DNA obtained from in vivo chromatin (left panels) or in vitro reconstituted chromatin (right panels). The best linear fits between log(ChIP-qPCR enrichment value) and tag count are shown.

ROC AUC values are generally similar whether nucleosome occupancies are obtained in vivo or in vitro (Figure 3A). Not surprisingly, the values are somewhat higher with in vivo chromatin than with in vitro reconstituted chromatin for most TFs (33/41, notably for Abf1 and Reb1). This indicates that some of the nucleosome depletion at binding sites is a consequence of TF binding and not really an effector of it. Nevertheless, the correlation between in vivo and in vitro values is remarkably good (R = 0.79; R = 0.90 if Abf1 and Reb1 are removed as outliers).

Neither the ROC AUC values nor the raw differences in tag counts used by Kaplan et al lend themselves to a simple interpretation in terms of the amount of TF binding information that lies in the intrinsic binding specificity of nucleosomes. To assess more directly how much of an effect on TF binding is encoded by the intrinsic DNA binding specificity of nucleosomes, we determined the apparent binding occupancies of 107 perfect consensus binding sites in the genome using ChIP-qPCR (Table S1). Between 16 and 33 consensus sites were assayed for each of four TFs (Dig1, Bas1, Gcn4 and Nrg1). The four TFs are typical of those evaluated in Figure 3A, lying close to the best fit through the data, but have associations with nucleosome occupancy that are skewed to lower-than-average values. This makes them particularly stringent targets for independent evaluation.

Figure 3B shows that ChIP enrichment values are correlated with nucleosome occupancy in the expected direction (i.e. higher ChIP enrichment is correlated with lower nucleosome occupancy). Correlation coefficients to the in vivo nucleosome data average 0.34 and range from 0.05 for Bas1 to 0.60 for Nrg1. All except Bas1 are significantly different than 0 (i.e., show a significant inverse correlation between ChIP enrichment values and nucleosome occupancy). ChIP-qPCR enrichment values appear to be correlated with in vitro nucleosome data as well, but poorly. (Figure 3B; right hand set of panels). For Gcn4, for example, only ∼1.7% (R^2^ = 0.13^2^) of the variance in ChIP enrichment values is explained by intrinsic nucleosome binding, as defined by reconstituted chromatin, while 16% (0.40^2^) of the variance is explained by nucleosome positions in vivo. Overall, for the four TFs we assayed, we estimate that only about 5% of the variance associated with nucleosome occupancy differences in vivo is due to intrinsic nucleosome positioning; the rest is a consequence of other effects that determine chromatin structure in vivo.

The fact that all four TFs show correlations in the expected direction between ChIP-qPCR enrichment and nucleosome occupancy attests to the sensitivity of this analysis because the ChIP-chip based ROC AUC values for these same TFs are only marginally different than the value expected by chance. It is possible that the ROC AUC values are underestimated due to the definition of unbound sites that we chose to use. We chose to use random sites selected from promoter regions (600bp 5′ to ORFs) thinking they would be more appropriate controls for the TF-bound sites, but the ROC AUC values obtained using this background are lower than what is obtained when sites randomly selected from throughout the genome are used instead (data not shown). A systematic underestimation of the true ROC AUC value would also explain why Nrg1 has an ROC AUC value below 0.5, implying a direct association between nucleosome occupancy and binding, even though our ChIP-qPCR analysis unambiguously shows the expected inverse correlation.

While absolute ROC AUC values should be interpreted with caution, comparisons of ROC AUC values are valid because each calculation was performed using the same set of unbound sequences as background. Since there are 41 TFs for which we have performed analyses using ChIP-chip data, we use those data and the ROC AUC metric for all subsequent analyses, rather than the correlation to ChIP-qPCR enrichment values, for which we have data for only four of the 41 TFs.

### Regional nucleosomal density is generally more relevant to TF binding than precise nucleosome position

As discussed above, most of the 41 TFs are modestly associated with nucleosome depletion in vivo (Figure 1C, Figure S3). This is consistent with the conclusion we reached previously using much lower resolution nucleosome occupancy data. [4] Those data were obtained from histone ChIP-chip experiments using DNA microarrays whose probes mostly corresponded to entire ORFs or intergenic regions. [1] The TF-bound regions were also low-resolution, having been obtained using the same microarray probes. [13] In the current analysis we use the same low resolution ChIP-chip data but the precise binding sites within those regions have now been inferred from motif analysis, as described by MacIsaac et al. [16] Thus, the TF binding data has effectively been made higher resolution through bioinformatics methods. The nucleosome occupancy data available now is truly higher resolution.

**Figure 4:**
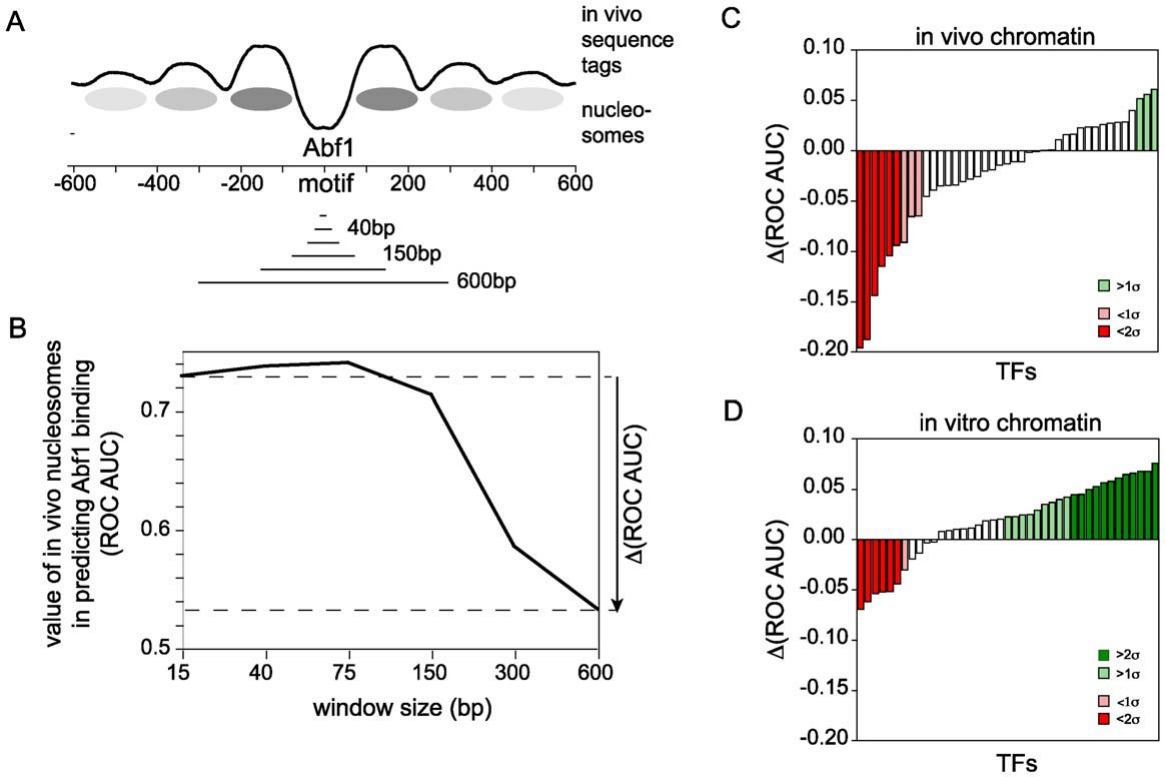
The effect of intrinsic sequence-dependent chromatin structure on TF binding is not dependent on exact nucleosome positioning. (A) Windowing scheme to simulate lower resolution data. Nucleosome tag counts around genomic loci (TF binding sites or control loci) were averaged over windows of 15, 40, 75, 150, 300 and 600bp as indicated by the lines at the bottom of the panel. Average tag counts around Abf1-bound sites are shown as in Figure 1A, along with the locations of nucleosomes inferred from those data. (B) ROC AUC values for the prediction of Abf1 binding sites based on low nucleosome occupancy, averaged over the indicated window sizes. The difference in ROC AUC in going from high resolution data (15bp) to simulated low resolution data (600bp) is indicated by the arrow. This measure of the effect of data blurring was used in panels C and D. (C) Effect of averaging in vivo nucleosome data on the correlation between nucleosome occupancy and TF binding for 41 TFs. Coloring of the histogram bars is based on the standard deviation of the values for abs(Δ(ROC AUC)), as indicated in the legend. (D) Same as panel C except that data from in vitro reconstituted chromatin was used rather than in vivo chromatin.

If transcription factor binding depends sensitively on the positioning of nucleosomes, we would expect high resolution data to produce a stronger association between nucleosome depletion and TF binding. To test this, we started with high-resolution data and simulated the effects of lower resolution data by averaging the high-resolution nucleosome occupancy data over windows of various sizes. Figure 4 (panels A and B) shows the effect of averaging these data on the association between nucleosome occupancy in vivo and Abf1 binding. Up to this point, we have used 15bp windows spanning TF binding sites (and randomly selected promoter sites) to calculate ROC curves and their areas. If a substantially larger window is used instead (150bp), the ROC AUC is noticeably lower; as the window is expanded to 300bp, the ROC AUC drops even more precipitously, and by 600bp, there remains only a very small association between low nucleosome occupancy and Abf1 binding sites. This dramatic drop in ROC AUC scores is expected because there are high occupancy nucleosomes flanking the Abf1 binding sites; a 300bp window includes the peaks of these nucleosomes, while a 600bp window includes all of those nucleosomes and a bit more of others. Window sizes of 40bp or 75bp actually show somewhat higher ROC AUC scores than the 15bp window, reflecting the fact that the average bound Abf1 has a nucleosome-depleted window that is about 50–75bp (Figure 4A).

We repeated this analysis for all 41 TFs, comparing the ROC AUC values obtained with 600bp windows to those obtained with 15bp windows (Figure 4C; Figure S4). Abf1 and Reb1, the two outliers in what is otherwise a good correlation between the effects of vivo chromatin vs. in vitro chromatin (Figure 3A), are exceptionally strongly affected by the averaging of nucleosome position data. This is because they have such a strong effect on local nucleosome density: displacement of nucleosomes at the binding site results in high nucleosome occupancy immediately adjacent to the site, and therefore there is a rapid regression to the mean nucleosome occupancy as the window size is expanded. Although there are exceptions, and the effect is less dramatic, this is a trend that is seen for the set of TFs as a whole. Altogether, a majority of TFs (∼60%) show poorer association with nucleosome occupancy when those occupancy values are averaged over 600bp. In addition, of the TFs that are most dramatically affected by averaging nucleosome occupancy over 600bp (i.e. those with changes greater than two standard deviations larger than the mean), all are adversely affected by the averaging of nucleosome occupancy.

Remarkably, the opposite effect is observed when in vitro reconstituted chromatin is used in the calculations rather than in vivo chromatin. An improvement in the association between binding and nucleosome occupancy is found for about three quarters of the TFs when in vitro nucleosome occupancies are averaged over 600bp. Among TFs that show the most dramatic effects (i.e. those exceeding the mean by 2σ), twice as many are improved as are made worse. At a cutoff of 1σ, three times as many are improved as are made worse. The number of TFs that are adversely affected by blurring of the in vivo data, and the number of TFs that are positively affected by blurring of the in vitro data, are each significantly different than the numbers expected by chance (p∼0.02).

The results with reconstituted chromatin are important because it is those data that are most relevant to an understanding of intrinsic nucleosome binding specificity and its effect on TF binding. The analyses of in vivo nucleosome data serve as a kind of computational control, showing that simulation of low resolution data does indeed weaken the association with TF binding, as would be expected if the precise nucleosome location, as defined by high-resolution sequencing experiments, were relevant to TF binding.

**Figure 5:**
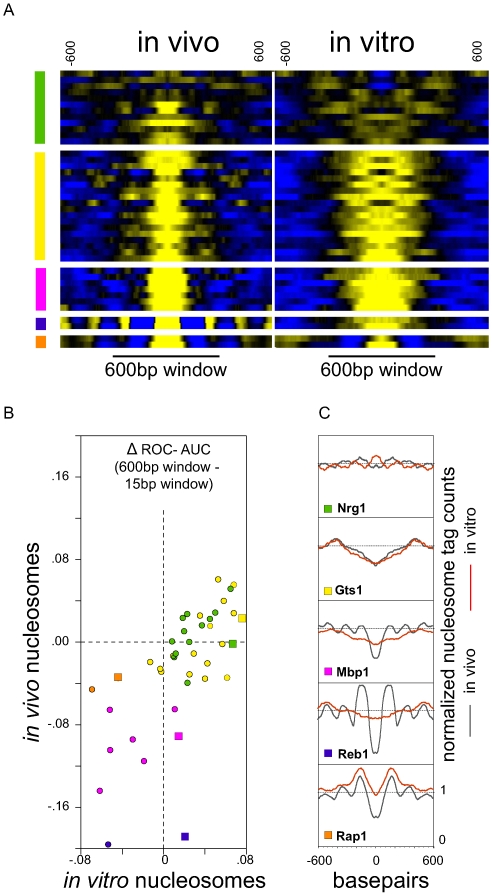
Clustering of nucleosome occupancy profiles suggests more than one reason for the efficacy of in vitro nucleosome occupancy blurring. (A) Heat map of the 1200bp nucleosome occupancy profiles surrounding TF binding sites. Each row represents the average profile around binding sites for one of the 41 TFs. The left side is based on the in vivo nucleosome map; the right is based on the in vitro nucleosome map. Tag counts were separately normalized to a mean of 0 for each of 1200bp in vitro and in vivo windows. Yellow represents low tag counts, and blue high. The TFs have been placed into five groups based on k-means clustering (Methods) and are ranked, within the groups, based on the improvement in association with TF binding when nucleosome occupancy is averaged over 600bp window rather than 15bp. Nucleosome-poorer regions are typically broader in the in vitro maps, and some TFs show nucleosome enrichment over TF binding sites in the in vitro maps. (B) Changes in ROC AUC values for the prediction of TF binding using blurred data (600bp vs 15bp). TFs are colored according to the clusters in which they fall based on the in vivo and in vitro nucleosome occupancy profiles surrounding their binding sites (note color key in panel A). For each group of TFs, the one with the greatest improvement using blurred in vitro nucleosome data is indicated by a square rather than a circle. (C) In vitro and in vivo nucleosome tag counts in a 1200 bp window surrounding bound sites. The five TFs shown are representative of the five profile clusters and are indicated by squares in panel B.

The question then, is why is there an improvement in the correlation between TF binding and nucleosome occupancy when high-resolution data for the in vitro nucleosome date are averaged so as to simulate lower resolution data? To investigate this question further, we examined more closely the patterns of nucleosome occupancy around TF binding sites in vivo and in vitro. To that end, we clustered the 41 TFs into five groups based on their in vivo and in vitro nucleosome profiles (Methods; Figure 5A), and confirmed that members of these groups share similarities in their sensitivity to data blurring in vivo and in vitro (Figure 5B). Most of the TFs for which the blurring of in vitro nucleosome data improves the association with binding have at least one of two properties in their nucleosome occupancy profiles that can explain this result: (i) the nucleosome poor region around the binding site is broad, such that averaging around the binding site provides greater contrast with random control sites or (ii) the binding site is actually higher in nucleosome density than the surrounding regions, so blurring the data encompasses flanking regions that are lower in nucleosome density. This latter set, in particular, suggest that the precise genomic position favored by nucleosomes is less relevant to TF binding than is overall nucleosome density in the region. This is perhaps the case because nucleosomes that occlude binding sites can be displaced to nearby regions at little energetic cost. In contrast, binding of Rap1/Fhl1 is correlated best with local nucleosome occupancy, and is adversely affected by blurring of the data. These TFs, unlike all others, tend to have well occupied nucleosomes that immediately flank their binding sites in vitro. As expected, TFs with similar patterns of nucleosome occupancy around their binding sites are also affected in similar ways by the averaging of nucleosome occupancy data (Figure S4).

### The averaging of high-resolution nucleosome occupancy data accentuates the effects of nucleosomes in a computational model of TF binding

As a further test of the effect of blurring high resolution nucleosome position data, we incorporated the data into a computational model that predicts TF binding to genomic regions. [18] The model uses position weight matrices (PWMs) to estimate K_d_ values for all sequence windows in the genome, and from those K_d_ values and an assumed protein concentration, it calculates the probability the protein is bound to at least one location within a genomic interval. Previously, we showed that low resolution nucleosome occupancy data, obtained from histone ChIP-chip experiments, could be used as weights in the calculation of K_d_ values in this model, and that these weights improved the prediction of binding as verified via a ChIP-chip experiment. [4] Others have also shown the utility of incorporating nucleosome occupancy data in this way. [19]

**Figure 6:**
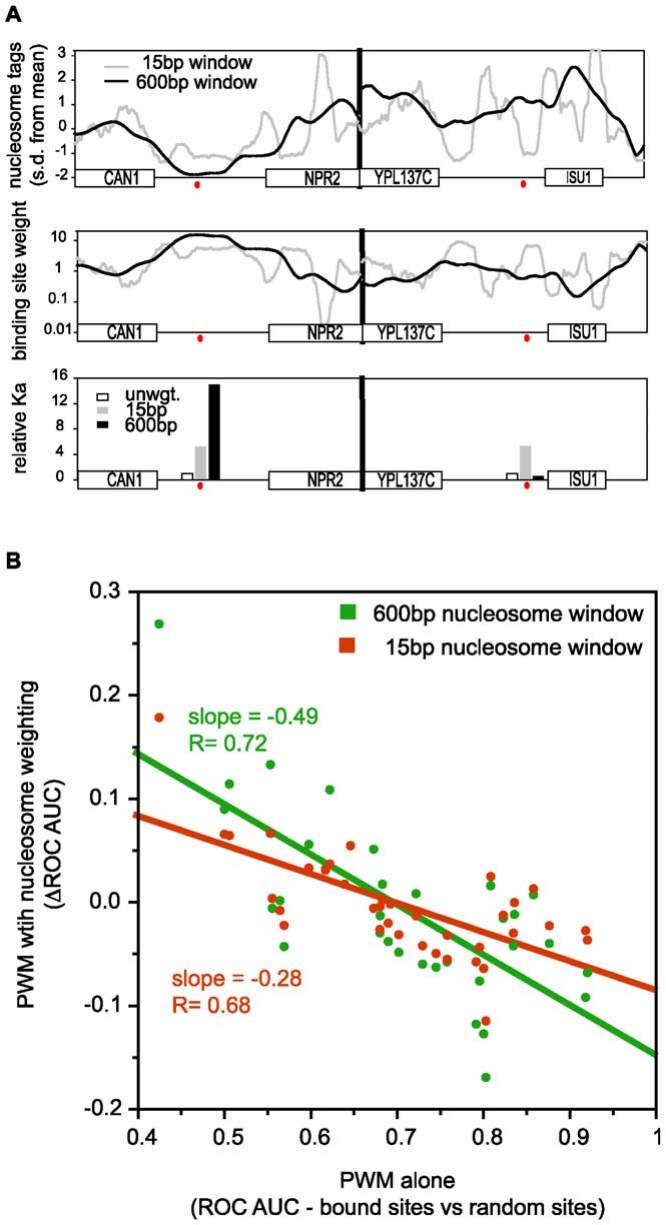
The blurring of intrinsic nucleosome occupancy data accentuates the effects of nucleosomes in a computational model of TF binding. (A) Illustration of how nucleosome occupancies are used to weight the predicted binding affinities of sequence motifs (top panel): Two 2.4kb genomic regions (CAN1/NPR2 and YPL137C/ISU1) showing normalized nucleosome tag counts from in vitro reconstituted chromatin, averaged over 15bp windows (gray line) or 600bp windows (black line). Red dots indicate the location of a perfect Gcn4 consensus site in each region. (middle panel): Same as the top panel except the lines show the conversion of normalized tag counts into weights that can be applied to Position Weight Matrix based estimates of TF binding affinity. Note that the weights are plotted on a log scale. Details of the weighting scheme are given in Methods. (bottom panel): Predicted equilibrium binding constants for the two sequence-identical Gcn4 sites (relative neighed K_a_ = 1; white histogram bars). High-resolution nucleosome data (15bp window; gray bars) increases the effective K_a_ of the two sites by about the same amount because the local nucleosome occupancy for both sets is about the same, and lower than average. Averaged over 600bp, the CAN1/NPR2 site is in a much lower-than-average nucleosome occupancy region while the YPL137C/ISU1 site is in a higher-than-average region. As a result, the predicted effective binding affinities of these two sites, subject to low resolution nucleosome occupancy (black bars) are very different. (B) Effect of nucleosome-based weighting on the prediction of TF bound promoters. Each dot is a TF. The value along x-axis shows how well the PWM, used in a computational model of TF binding, predicts which promoters are bound. This is quantified as the area under an ROC curve (ROC AUC). Plotted against this value is the change in ROC AUC that is obtained by weighting genomic loci on the basis of high-resolution nucleosome data (orange; 15bp window) or on the same data averaged over 600bp windows.

Here, we used the same weighting function and parameter values developed previously, but instead of applying weights based on large genomic regions, we used the base-pair resolution, in vitro nucleosome position data of Kaplan et al. [9] The ChIP-chip data of Harbison were used to evaluate the predictive efficacy of these weights. [13] Specifically, we first scored yeast promoters for the probability of binding based on our computational model and the PWMs for transcription factors. [18] We then evaluated how well this predicted binding identified genes whose promoters are bound experimentally. The calculations were then repeated twice, once using weights based on in vitro nucleosome occupancy data at 15bp resolution, the other using weights based on averaging this nucleosome occupancy data over sliding 600bp windows. Figure 6A illustrates the effect of these weighting schemes on two genomic regions, each containing a perfect consensus binding site for Gcn4.

For each TF, we obtained three ROC AUC values that express how well binding is predicted: one based on the PWM alone; the second based on the PWM, but with genomic position weights determined by high resolution nucleosome position data; and the third based on the PWM and weights determined by simulated low resolution nucleosome position data (i.e. high resolution data averaged over 600bp windows). For TFs whose binding is well predicted by genomic sequence and the PWM alone, the inclusion of weights based on nucleosome occupancy evidently adds noise to the calculation, worsening the predictions. However, for TFs whose binding is poorly predicted by sequence alone, the inclusion of binding affinity weights can substantially improve the prediction of binding (Figure 6B).

Strikingly, the effect of intrinsic nucleosome position data on binding predictions is accentuated with the simulated low resolution data. This is the opposite of what we would expect if precise nucleosome positioning were typically of great relevance to the binding of transcription factors, and it is the opposite of what we observed in most cases with the in vivo data. Of course, the improvement in binding predictions with blurred data is for the set of bound promoters as a whole; within this set, some of the promoters bound by the TF fall in rank even if, overall, weighting improves the ROC AUC value (Figure S5). For example, even though Yap5 is the most responsive TF to nucleosome averaging overall, 22 of the 88 promoters bound by Yap5 drop in rank when in vitro nucleosome data is averaged over a 600bp window. Nevertheless, the overall effect of blurred nucleosome data on binding predictions provides additional support for our contention that it is the regional nucleosome occupancy that matters most in the localization of TF binding sites, not the precise position of the nucleosome.

## Methods

### Datasets

The yeast genome sequence (Aug 2008 build) and gene feature files were obtained from the Saccharomyces Genome Database (SGD). [20] Promoter sequences were defined as the 600bp 5′ to the start of transcription of protein coding gene. The genomic positions of ChIP microarray probes and their ChIP enrichment p-values using different epitope tagged transcription factors under normal growth conditions (YPD media, 30°C) were obtained from Harbison et al. [13] The nucleosome sequence tag maps of Kaplan et al. [9] were obtained from GEO (accession number GSE13622). Sequence coordinates for each data set were converted to match the SGD Aug 2008 version of the genome based on a list of coordinate differences maintained at SGD. Genomic loci deemed to be potential transcription factor (TF) binding sites on the basis of sequence analyses were obtained from MacIsaac et al. [16]. For each potential TF binding site, the ChIP-chip probe spanning that site was identified and the ChIP enrichment p-value for that that probe was then assigned to the binding motif. Binding motifs with p-values<0.001, were classified as being bound by their respective TFs. 41 TFs had ≥50 bound motifs by this criterion and were used for all subsequent analyses. For the analysis of Abf1 binding, Abf1 motifs were further binned into p-value intervals 0.001–0.01, 0.01–0.1, 0.1–0.5, >0.5.

### Mapping nucleosome sequence tag counts to transcription factor binding motifs

The nucleosome sequence tag data provided by Kaplan et al consists of a 5′ end, determined by sequencing, and a 3′ end 146bp away that is based on knowledge about the size of nucleosomes and on the preparation in the experiment of ∼150bp sized DNA by nuclease treatment and size-selection. [9] For simplicity, we refer throughout the paper to the inferred 146bp sequence as a ‘nucleosome tag’, or simply a ‘tag’. The number of tags spanning a particular genomic basepair can be enumerated and is taken to be a measure of the nucleosome occupancy at that basepair. For most analyses, tag counts were averaged over windows that were centered on a binding motif or on randomly selected basepairs from within promoters. We refer to the windows as being of size 15, 40, 75, 150, 300 and 600 bp, though technically some of the windows are 1bp longer depending on whether the motif is of even length or odd.

### ROC analysis

Receiver operating characteristic (ROC) curves and the area under those curves (AUC) were used to quantify the ability of a predictor (nucleosome tag counts) to correctly classify sequences as TF bound (defined by ChIP-chip) or unbound (randomly selected from yeast gene promoters). Where error bars are shown for ROC AUC values, these were estimated from 1000-fold bootstrap re-sampling. ROC AUC values were also used to quantify the predictive value of a TF binding prediction algorithm, with and without weights based on nucleosome occupancies. In this case, the predictor is the estimated TF binding occupancy and the question is how well that value classifies promoters as TF bound or unbound. Unbound sites were selected from 1000 randomly picked yeast promoters, defined as the 600bp region 5′ to the start of a gene.

### Clustering of nucleosome profiles

For each of the 41 TFs, and for each of the two nucleosome datasets, we enumerated the number of nucleosomal sequences spanning each basepair in a 1200bp window. The tag counts were averaged across the center of the profiles, and normalized to the mean value in that profile. These 1200bp normalized windows were used to visualize the profiles for each TF (Figure 5). Clustering was performed based on catenation of the values for the central 600bp from the in vivo data and the central 600bp from the in vitro data. Similarity in profiles was defined by the Pearson correlation coefficient, and the clusters were identified by k-means clustering. A value of k = 5 is shown based on subjective assessment of the clusters for different values of k.

### TF binding occupancy predictions and nucleosome weighting

The computer program GOMER was used to calculate predicted binding affinities in yeast promoters based on TF-specific position weight matrices (PWM) and, optionally, affinity-modifying weights that were applied to genomic regions based on nucleosome tag counts. [18] Genes with bound promoters were defined by Harbison et al based on binding to 5′ intergenic regions in normal (YPD) conditions.[13] PWMs were obtained from the work of MacIsaac et al. [16] Of the 41 TFs we studied, PWMs were available for 36 and were used for this analysis. For the purpose of calculating and applying nucleosome occupancy weights to genomic subsequences, we subdivided the genome into non-overlapping 15bp segments. The local nucleosome occupancy for each segment was defined by the average tag count within that segment, and the regional nucleosome occupancy for the segment was defined as the average tag count in the 300bp spanning the segment. The effect of nucleosome occupancy on predicted TF binding was defined essentially as described earlier for histone ChIP-chip data. [4] Predicted K_a_ values were modified at each site according to the nucleosome tag count in that region,
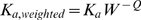
where **W** is a weighting parameter and **Q** is the tag count expressed as the number of standard deviations above zero. Zero tags was used as the reference, rather than the mean hybridization intensity used in our earlier work, so that regions that had no mapped nucleosomes for technical reasons (e.g. non-unique sequences in the genome) were given weights of 1. Note that higher nucleosome occupancies result in exponentially lower predicted affinities for the TF. A value of 4 was used for the weighting factor, **W**, based on the parameterization of this value in earlier work using low-resolution histone ChIP-chip data. We chose to fix this value rather than fitting it to the new data.

### Chromatin immunoprecipitation and quantitative PCR of consensus binding sites

Yeast strains expressing TAP-tagged transcription factors BAS1, DIG1, GCN4 and NRG1 were obtained from Open Biosystems. For each TF, we identified a set of perfect consensus binding sites that lay within genomic regions enriched in the ChIP-chip experiments of Harbison et al. [13] PCR primers were designed flanking each of these sites, generating amplicons of 100–150bp. Chromatin immunoprecipitation was carried out essentially as described. [4] Briefly, yeast cells were grown to late log phase, fixed with 1% formaldehyde for 15 minutes at 30°C and then quenched with a final concentration of 125mM glycine. Cells were disrupted with glass beads in lysis buffer (50 mM HEPES-KOH pH 7.5, 300 nM NaCl, 1 mM EDTA, 1.0% Triton X-100, 0.1% sodium deoxycholate) and the extract sonicated to an average size of ∼500bp. Immunopurification was carried out with Sepharose-6 Fast Flow IgG beads as described by the manufacturer (GE Healthcare). Input DNA and immunoprecipitated DNA were treated with RNaseA and ProteinaseK, and purified by phenol-chloroform extraction. DNA was quantified by qPCR, using two control sequences that lack similarity to the binding motifs of any of the TFs studied. Three or more biological replicates were performed for each transcription factor and multiple technical replicates were performed for most sites and for most biological replicates. The enrichment values we report for a binding site are based on the arithmetic mean of the ΔΔCt values using input DNA and the average of the two control sites for comparison. Not all sites were bound in our assays despite being consensus sites selected from genomic regions reported to be bound in ChIP-chip experiments. For purposes of Figure 3, sites with nominal enrichment values of less than 1 were changed to 1. Also, four sites that lie in regions of exceptionally high nucleosome tag counts (>>600) were plotted as having values of 600.

### Data and software availability

A list of the sites assayed by ChIP-qPCR and their enrichment values is available as supplementary material. The GOMER program has been described previously and is made freely available from the authors on request. [18]

## Discussion

We have confirmed the conclusion of Kaplan et al [9] that sequence-specific binding of nucleosomes plays a role in the selection of binding sites by TFs, although most TFs are more strongly associated with in vivo nucleosome positions than in vitro. This reflects the fact that TF binding itself is one of the causes of the differential nucleosome occupancy in vivo that is correlated with TF binding. The stronger association with in vivo nucleosome data was even more evident in the experiments we performed using ChIP-qPCR enrichment at consensus binding sites. The relatively weaker association with nucleosome binding in vitro perhaps lies in the different standards being applied in the two analyses. In the ChIP-chip analyses we asked how well nucleosome occupancy could classify bound vs. unbound sites but in the ChIP-qPCR experiments, we assessed quantitatively the correlation between nucleosome occupancy and TF ChIP enrichment. Perhaps it is too much to expect strong correlations between ChIP enrichment values and nucleosome occupancies as there are many factors that contribute to ChIP enrichment. Indeed, it is not even clear how strong the correlation is between TF occupancy and ChIP enrichment.

The difference in TF binding associations for the in vivo and in vitro nucleosome data is most striking for the outliers Abf1 and Reb1. These two TFs are thought to play key roles in chromatin remodeling and the formation of nucleosome free regions (NFRs) in yeast promoters [21]. The association between binding and nucleosome depletion in vivo is so strong for these factors that we find clear evidence of Abf1-mediated depletion even in genomic regions for which the ChIP-chip enrichment p-value is far worse than what can be considered significant. This is not unexpected because binding is not a discrete phenomenon that lends itself to absolute cutoffs, and it has been shown that authentic and biologically relevant binding occurs even for sequences whose ChIP enrichment p-values are extremely poor. [14] We provide further evidence for this conclusion by showing that low nucleosomal occupancies are predictive of Abf1 binding, even when the statistical confidence in binding is extraordinarily low.

The most important question we have addressed in this paper is the following: how much does the preferred location of nucleosomes matter to the selection and occupancy of binding sites by transcription factors. The way we sought to answer this is to ask whether data resolution is important to the conclusion that TF binding is associated with low nucleosome occupancy regions. Lower resolution data was simulated by averaging the high resolution data over increasingly larger windows. If the precise location and occupancy of nucleosomes is of great importance for the binding of TFs, then lower resolution data ought to do a worse job of showing the relationship between nucleosome occupancy and TF binding. We find that this is generally true for the in vivo data, which is determined in part by TF binding. However, just the opposite is true for the in vitro nucleosome data: simulating low resolution data by averaging over 600bp windows generally improves the predictions. This could not have been the result if mapped nucleosome positions were both accurate and strongly preferred over alternative positions.

For three reasons, we believe the resolution to this observation lies not in questioning the accuracy of the nucleosome occupancy maps, but in the assumption that the precision of intrinsic nucleosome binding matters a great deal to where transcription factors bind. First, the methodology for mapping nucleosomes was the same in vivo and in vitro. The in vivo maps show the expected trend, wherein a blurring of the data weakens the correlations, and we know of no reason to expect or believe that the accuracy of nucleosome mapping is different in the two different chromatin preparations. Second, the energetic differences between a preferred nucleosome configuration and an alternative are not expected to be large, in general. [22],[23] This is especially true if the overall nucleosome occupancy is low because then there are many alternatives that accommodate transcription factor binding, increasing the configurational entropy of the binding-tolerant alternative(s). Lower nucleosome density also allows each nucleosome to find local positions that are closer to the optimal. Third, the data itself suggests that most preferred nucleosome occupancies are only slightly more favorable than alternatives because the tag count densities under well-occupied nucleosomes are typically only a few fold higher than they are in the adjacent spacer regions. This suggests a free energy difference between preferred position and the spacer region on the order of 1 kcal/mol or less. The high tag count in spacer regions might reflect limitations in the experimental method, but it is also consistent with our expectations based on the energetics of nucleosome binding. [22],[23] We conclude that intrinsic nucleosome binding specificity plays a role in determining the selection and occupancy of transcription factor binding sites with which nucleosomes compete. However, the role is not so much in occluding binding sites based on precise nucleosome positioning, but more in defining broad regions of lower or higher nucleosome density that accommodate TF binding with differing degrees of ease.

There are several exceptions to the rule that the blurring of in vitro nucleosome data improves the association between nucleosome occupancy and binding. These exceptions tend to be TFs like Fhl1 and Rap1 that have relatively high nucleosome density flanking their binding sites. There is also a modest tendency for these TFs to be bound less frequently at TATA+ promoters (15±6% vs 27±9%; Figure S6). However, it is not clear whether there is a mechanistic connection between these two facts.

The second important observation we report is the difference between nucleosome maps constructed in the conventional way, from uncrosslinked chromatin, and those constructed from formaldehyde-crosslinked chromatin. This difference is not only well correlated with TF binding but is, if anything, better correlated than nucleosome occupancy in the uncrosslinked sample.

The origin of this effect is uncertain. Conceivably it is a consequence of differences in higher order chromatin around TF binding sites, or it may be that the crosslinked TF itself provides protection against nuclease digestion. However, both explanations would require that at least some of the protected DNA survive size selection for mononucleosome-sized DNA. Another problem with the TF-protection explanation is that we would expect higher TF concentrations to increase the difference between crosslinking and non-crosslinking at binding sites, whereas the opposite is true, at least for Gal4 binding at the GAL1–GAL10 promoter (Figure S2).

Alternatively, and more simply, the excess sequence tags may be due to transient nucleosomes sitting on TF binding sites. Crosslinking might be expected to have the greatest effect on nucleosomes that are ‘volatile’ relative to other nucleosomes in the genome: nucleosomes with slow association/dissociation kinetics (slow relative to nuclease treatment) should be relatively unaffected by crosslinking, while nucleosomes with fast kinetics should have their apparent occupancies increased because of the ease with which nucleosomes can be crosslinked to DNA. Competition with TFs can be expected to alter the apparent kinetics of nucleosomes by competing with them for reassociation, and histone turnover measurements have indeed shown faster exchange kinetics at yeast promoters [24] and at presumptive regulatory elements in Drosophila. [25] It may also be that the nucleosomes at binding sites contain histone variants that render their binding inherently more labile.[26] However, we were unable to establish an association between the nucleosome difference map (effect of crosslinking) and the replication-independent exchange rate of nucleosomes mapped at 265bp resolution (data not shown). [24]

Whatever the mechanisms, it seems clear that there is an association between regions of regulatory protein binding and higher nucleosome lability. The crosslink-noncrosslink difference map, which seems to be identifying labile nucleosomes, might therefore be used to discover non-histone protein binding sites in the genome.

The interactions among nucleosomes, transcription factors, and the enzymes that act on DNA and chromatin are complicated, but central to a deep understanding of gene regulation. Nucleosomes are a dominant factor in these interactions because they cover roughly 80% of the genome. Together with their intrinsic DNA sequence specificity, this adds further complexity to the problem. Our analyses suggest a simplifying principle to this complexity, namely that the precise position defined by nucleosome sequence specificity is not (on average, and for most TFs) of critical importance. Instead, the genome has evolved to define regions of lower and higher intrinsic nucleosome occupancy and these broad regions typically matter more than the precise most-favored configuration. Having said that, we expect there will be many exceptions in which precise positions are proven to be important. The technology now exists to explore these phenomena in greater detail and to begin to examine the kinetics of remodeling from one chromatin state to another. As data accumulates, we are confident that the incorporation of DNA-encoded nucleosome position information into computational models of TF binding will continue to improve the predictive quality of these models.

## Supporting Information

Table S1 Tab-delimited ChIP-qPCR data used in Figure 3.
(0.01 MB TXT)

Figure S1 Crosslinking of chromatin generally weakens the association between low nucleosome density and TF binding. ROC AUC values for 41 TFs are generally lower when crosslinked chromatin data is used rather than uncrosslinked.
(0.73 MB EPS)

Figure S2 Genome browser tracks showing nucleosome tag counts in the GAL1–GAL10 regions for cells grown in YPD or in galactose-containing media. All data are from Kaplan et al (ref 9). Profiles are shown for crosslinked chromatin, uncrosslinked chromatin, and the difference between the two. A prominent peak in the difference map is apparent in YPD conditions, but is less prominent in galactose, presumably because Gal4 binding reduces the occupancy of the occluding nucleosome. The existence of the difference peak in YPD implies the existence of an unusually labile nucleosome over the Gal4 binding sites. Gal4 is known to have some occupancy of its GAL1–GAL10 promoter sites even in glucose, so the apparent lability of this nucleosome probably reflects modest competition from Gal4.
(2.02 MB EPS)

Figure S3 Nucleosome occupancy profiles around TF binding sites for all 41 TFs with at least 50 bound sites, and for the randomly selected promoter sites used as controls. Nucleosome profiles in black are from the in vivo data; profiles in red are from the in vitro data (ref 9). In vivo and in vitro data were each normalized genome-wide to a mean value of 1, and symmetrized around the center of the binding sites. The TFs are organized into color-coded blocks based on the k-means clusters of Figure 5. Within the blocks, the TFs are arranged in ascending order based on ΔROCAUC (in vivo) - ΔROCAUC (in vitro). The slight dip in the profiles for the random promoter sites (bottom panel) reflects the fact that promotes tend to have lower nucleosome occupancy than transcribed regions.
(4.46 MB EPS)

Figure S4 Window-size dependency of nucleosome-TF binding correlations and the relationship to nucleosome occupancy profiles. (A) The difference in ROC AUC values at different window sizes was used to cluster TFs by k-means clustering. k = 5 was chosen to mirror the clustering of TFs by nucleosome occupancy profiles (Figure 5). The identities of the TFs in each cluster are shown at the right, along with a color-coded identification of the cluster to which the TF belongs based on nucleosome occupancy profiles. (B) Nucleosome occupancy profiles. This panel is identical to the one shown in Figure 5, but is reproduced here to facilitate comparisons. As described in the text, there is a rough congruence to the clustering, which reflects the fact that window-size effects are a consequence of the distribution of nucleosome occupancy around the binding sites.
(5.02 MB EPS)

Figure S5 Not all binding sites or promoters are affected in the same way by data blurring. (A) Gst1 is the TF that shows the strongest positive effect of data blurring of the in vitro transcription data (fig. 4). Each Gst1 binding site is ranked relative to all the other Gst1 sites, along with the 1000 random promoters site controls, and the normalized rank (1: best, 0:worst) is plotted using a 600bp window vs. a 15bp window. Most, but not all, sites improve in rank with data blurring. Normalized ranks are used rather than absolute ranks in this case because absolute ranks depend on the number of control sites used, which is arbitrary. (B) Similar analysis to B, but based on the effect of nucleosome-based weighting on the prediction of TF binding. Yap5 is the TF most improved by averaging nucleosome occupancy prior to applying those values to the genomic sequences as a way of weighting binding sites (fig. 6). Most, but not all, promoters improve in rank when nucleosome occupancy is averaged over 600bp rather than using the local occupancy. Note that ranks are plotted on a log scale.
(0.79 MB EPS)

Figure S6 For the minority of TFs whose association between binding and nucleosome-poor regions is worsened by blurring of the nucleosome occupancy data (circles lying below the diagnonal), there is a slight tendency for those TFs to also be bound less frequently to TATA-containing promoters (lighter color). However, the effect is small and it is likely that any connection between the two properties is indirect.
(0.43 MB EPS)

## References

[1] Lee CK, Shibata Y, Rao B, Strahl BD, Lieb JD (2004). Evidence for nucleosome depletion at active regulatory regions genome-wide.. Nat Genet.

[2] Yuan GC, Liu YJ, Dion MF, Slack MD, Wu LF (2005). Genome-scale identification of nucleosome positions in S. cerevisiae.. Science.

[3] Pokholok DK, Harbison CT, Levine S, Cole M, Hannett NM (2005). Genome-wide map of nucleosome acetylation and methylation in yeast.. Cell.

[4] Liu X, Lee CK, Granek JA, Clarke ND, Lieb JD (2006). Whole-genome comparison of Leu3 binding in vitro and in vivo reveals the importance of nucleosome occupancy in target site selection.. Genome Res.

[5] Miller JA, Widom J (2003). Collaborative competition mechanism for gene activation in vivo.. Mol Cell Biol.

[6] Drew HR, Travers AA (1985). DNA bending and its relation to nucleosome positioning.. J Mol Biol.

[7] Satchwell SC, Drew HR, Travers AA (1986). Sequence periodicities in chicken nucleosome core DNA.. J Mol Biol.

[8] Peckham HE, Thurman RE, Fu Y, Stamatoyannopoulos JA, Noble WS (2007). Nucleosome positioning signals in genomic DNA.. Genome Res.

[9] Kaplan N, Moore IK, Fondufe-Mittendorf Y, Gossett AJ, Tillo D (2008). The DNA-encoded nucleosome organization of a eukaryotic genome.. Nature.

[10] Richmond TJ, Davey CA (2003). The structure of DNA in the nucleosome core.. Nature.

[11] Mavrich TN, Ioshikhes IP, Venters BJ, Jiang C, Tomsho LP (2008). A barrier nucleosome model for statistical positioning of nucleosomes throughout the yeast genome.. Genome Res.

[12] Yuan G, Liu J (2008). Genomic sequence is highly predictive of local nucleosome depletion.. PLoS Comput Biol.

[13] Harbison CT, Gordon DB, Lee TI, Rinaldi NJ, MacIsaac KD (2004). Transcriptional regulatory code of a eukaryotic genome.. Nature.

[14] Tanay A (2006). Extensive low-affinity transcriptional interactions in the yeast genome.. Genome Res.

[15] Clarke ND, Granek JA (2003). Rank order metrics for quantifying the association of sequence features with gene regulation.. Bioinformatics.

[16] Macisaac KD, Wang T, Gordon DB, Gifford DK, Stormo G (2006). An improved map of conserved regulatory sites for Saccharomyces cerevisiae.. BMC Bioinformatics.

[17] Giresi PG, Kim J, McDaniell RM, Iyer VR, Lieb JD (2007). FAIRE (Formaldehyde-Assisted Isolation of Regulatory Elements) isolates active regulatory elements from human chromatin.. Genome Res.

[18] Granek JA, Clarke ND (2005). Explicit equilibrium modeling of transcription-factor binding and gene regulation.. Genome Biol.

[19] Narlikar L, Gordân R, Hartemink AJ (2007). A nucleosome-guided map of transcription factor binding sites in yeast.. PLoS Comput Biol.

[20] Cherry JM, Adler C, Ball C, Chervitz SA, Dwight SS (1998). SGD: Saccharomyces Genome Database.. Nucleic Acids Res.

[21] Hartley P, Madhani H (2009). Mechanisms that specify promoter nucleosome location and identity.. Cell.

[22] Gottesfeld JM, Luger K (2001). Energetics and affinity of the histone octamer for defined DNA sequences.. Biochemistry.

[23] Lowary PT, Widom J (1997). Nucleosome packaging and nucleosome positioning of genomic DNA.. Proc Natl Acad Sci USA.

[24] Dion MF, Kaplan T, Kim M, Buratowski S, Friedman N (2007). Dynamics of replication-independent histone turnover in budding yeast.. Science.

[25] Mito Y, Henikoff JG, Henikoff S (2007). Histone replacement marks the boundaries of cis-regulatory domains.. Science.

[26] Jin C, Zang C, Wei G, Cui K, Peng W (2009). H3.3/H2A.Z double variant-containing nucleosomes mark ‘nucleosome-free regions’ of active promoters and other regulatory regions.. Nat Genet.

